# The central role of pathology labs in breast cancer precision oncology: a call for action

**DOI:** 10.1038/s41523-023-00506-5

**Published:** 2023-01-25

**Authors:** Giancarlo Pruneri, Daniele Lorenzini, Mauro G. Mastropasqua, Giuseppe Perrone, Antonio Rizzo, Donatella Santini, Chiara C. Volpi, Saverio Cinieri, Alberto Zambelli, Anna Sapino, Isabella Castellano

**Affiliations:** 1 Fondazione IRCCS, Istituto Nazionale Tumori, Milan, Italy; 2 University of Milan, School of Medicine, Milan, Italy; 3 Department of Emergency and Organs Transplantation, Section of Anatomic Pathology, School of Medicine, University of Bari “Aldo Moro”, Bari, Italy; 4 Research Unit of Pathology, Campus Bio-Medico University, Rome, Italy; 5 U.O. Anatomia Patologica, Humanitas Istituto Clinico Catanese, Catania, Italy; 6 Pathology Unit, IRCCS, Azienda Ospedaliero-Universitaria di Bologna, Bologna, Italy; 7 Medical Oncology Division and Breast Unit, Senatore Antonio Perrino Hospital, ASL Brindisi, Brindisi, Italy; 8 Medical Oncology and Hematology Unit, IRCCS Humanitas Research Hospital, Rozzano-Milan, Italy; 9 Candiolo Cancer Institute, FPO-IRCCS Candiolo, Candiolo, Italy; 10 Department of Medical Science, University of Turin, Turin, Italy

**Keywords:** Breast cancer, Predictive markers

## Abstract

Multigenic tests represent an essential tool for the selection of adjuvant therapy in estrogen-positive/HER2-negative (ER + /HER2-) early breast cancer (BC). The workflow of these tests, either if they are externalized or carried out in-house, generates a workload for the pathology laboratories, that is often underestimated and may affect timely therapy initiation. Here, we describe the evolving role of pathology laboratories in using multigenic tests and, more in general, in providing adequate tissue for molecular analyses. Moreover, we propose a “reflex testing” model, in which pathologists, based on pre-specified and shared criteria, are expected to action multigene testing independently of multidisciplinary team discussion in ER + /HER2- BC patients, in order to optimize turnaround time and proper therapy intervention.

Breast cancer (BC) represents the most common malignant tumor in women, with approximately 2.3 million and 520,000 new cases per year occurring worldwide and in Europe, respectively, more than 90% of which are diagnosed at early stages (eBC)^1,2^. Approximately 70% of eBC are estrogen receptor (ER) positive and HER2 negative (ER + /HER2-), as assessed by immunohistochemistry (IHC) and, in HER2 IHC equivocal cases (2 + according to ASCO/CAP guidelines), by in situ hybridization (ISH) techniques within pathology units accredited by national health systems (NHS). In a large fraction of ER + /HER2- eBC patients, the potential benefit of adding chemotherapy (CHT) to endocrine therapy (ET) remains uncertain using traditional clinicopathological predictive and prognostic parameters, including age, menopausal status, clinical stage, levels of ER and progesterone receptor (PgR) immunoreactivity, nuclear grading and Ki-67 labeling index^3^. In this scenario, molecular assays have been developed to better define ER + /HER2- eBC patient prognosis or, as far as Oncotype Dx^®^ is concerned, to predict CHT benefit, eventually optimizing adjuvant treatment and sparing unnecessary CHT. The Oncotype Dx^®^ recurrence score (RS), generated by a 21 genes reverse-transcriptase-polymerase-chain-reaction (RT-PCR) assay running in formalin fixed-paraffin embedded (FFPE) specimens, has been demonstrated to outperform traditional clinicopathological criteria in identifying ER + /HER2- early BC patients with a very favorable outcome, who can therefore safely spare CHT^4^. Recent data from the randomized prospective clinical trials TAILORx and RxPONDER provided evidence that Oncotype Dx^®^ RS is predictive of CHT benefit in node-negative and positive (N1a) patients, respectively^5,6^. MammaPrint® is a microarray-based gene-expression signature of 70 genes, which was originally shown to be prognostic in a cohort of 295 patients^7^. The prospective randomized MINDACT trial recently showed that patients with a high-risk disease according to clinical variables, but with a low-risk molecular signature, had minimal, if any, benefit in adding CHT to ET^8^. Prosigna^®^ is a multigenic test based on the PAM50 gene signature^9^ that can predict a risk of recurrence (ROR) up to 10 years after surgery, identify the underlying biology and allow to classify BC intrinsic subtypes^10,11^. Finally, Endopredict^®^ (EP) is a quantitative RT-PCR assay evaluating eight cancer related genes predicting ROR in ER + /HER2- BC patients. Combining EP score with tumor size and node status (EPclin) resulted in a higher prognostic accuracy^12,13^. A recent comparative analysis carried out in the trans-ATAC cohort showed that Oncotype Dx® RS is mainly affected by estrogen module, whereas Prosigna^®^ ROR is dependent on proliferative information^14^. A recent update of the American Society of Clinical Oncology (ASCO) guidelines strongly recommended the use of Oncotype Dx^®^ in pre- and postmenopausal (>50 yrs) women with early stage, ER + /HER2- node-negative BC to guide decision on adjuvant CHT. For 1–3 node positive (N1) disease, the test is strongly recommended only in postmenopausal/ >50 years patients^15^. The other genomic tests have a lower level of evidence according to ASCO: in particular, Mammaprint^®^ is indicated only for patients with clinical high-risk disease (as previously described in the MINDACT trial), whereas Ki67/IHC should be used only when genomic tests are unavailable. Similar recommendations were released by the Cancer Care Ontario guidelines^16^. Furthermore, the European Commission Initiative on Breast Cancer recommended the use of Oncotype Dx^®^ RS in ER + /HER2-, N0 BC patients to assess the benefit of adjuvant CHT, whereas the Mammaprint^®^ 70-genes risk score should be used only in clinical high-risk patients as defined in MINDACT trial^17^. Along this line, the European Society of Medical Oncology (ESMO) guidelines on eBC suggest using multigenic tests in case of uncertainty regarding indications for adjuvant CHT, with the highest grade of recommendation (A) for Oncotype Dx^®^ and Mammaprint^®^^18^. The Italian Association for Medical Oncology (AIOM) updated guidelines (2021) state that multigenic tests are indicated when there is uncertainty regarding CHT benefit based on clinical, radiological and histopathological data, underlying the strong evidence (level 1a) provided by Oncotype Dx^®^^19^.

In 2021, two years after the approval for reimbursement by Lombardy regional authorities, a resolution of the Italian Ministry of Health established that commercially available multigenic tests should be offered to ER + /HER2 − eBC patients with an intermediate clinicopathological risk (Table 1). In Europe, multigene tests are reimbursed by NHS or, as for example in UK and Germany, by combining private and public funding. Furthermore, current guidelines for multigene test reimbursement in many European countries are limited to patients for which the traditional clinicopathological risk assessment cannot clearly establish the benefit of delivering CHT in combination with ET^18^. According to these criteria, about 10,000 eBC patients per year are expected to be tested by multigenic assays in Italy, nearly a half of which will eventually avoid CHT^20^.

**Table 1:** Italian criteria for multigenic prognostic test in breast cancer.

Low risk	High risk
All criteria must be satisfied (5/5)	Four criteria must be satisfied (4/5)
• Histological grade 1	• Histological grade 3
• Stage pT1a,b^a^	• Stage pT3/pT4
• Ki67 < 20%	• Ki67 > 30%
• ER > 80%	• ER < 30%
• No nodal metastasis (pN0)	• Nodal metastasis (pN + )^b^

^a^in case of pT1a the test is not indicated if two other criteria are satisfied.

^b^in case of more than three lymph node metastasis, test is not indicated.

Within universalistic health systems, multigene assays have therefore been conceived as second level tests to be actioned after, and fully integrated with, the traditional diagnostic algorithm. In this context, proper histological grading, pathological staging (tumor size and lymph node status), as well as the assessment of predictive/prognostic biological features (ER, PgR, Ki-67, HER2) by IHC and ISH assays represent pre-analytical steps of multigene testing, engaging pathology labs to reshape the diagnostic workflow for ER + /HER2- eBC patients. Multigene tests can be either externalized or run in house on dedicated platforms: in particular, Oncotype Dx^®^ requires sending slides to a central lab, while Prosigna^®^ and EP^®^ tests can also be performed in house by properly equipped labs. Either way, all multigene tests require an additional workload for pathology units, in terms of procedures, personnel time (lab technicians, pathologists, administrative staff), and reagents (tissue processing, slide cutting). The authors find surprising that these activities have not yet been fully valued and supported by NHS: for example, the IHC characterization with ER, PgR, HER2 and Ki-67 antibodies of BC patients is still not reimbursed in Italy^21^, an approach impinging proper staffing and the use of validated reagent kits in favor of less expensive lab developed tests. This could lower the overall quality and reproducibility of IHC analysis, also affecting the proper selection of cases to be profiled by multigene testing. Furthermore, precision oncology is increasingly reliant on different comprehensive genomic techniques for a precise identification of several clinically actionable molecular biomarkers in all cancer settings: as a result, pathology dedicate personnel, reagents and resources in collecting, preparing, and shipping tissue samples to public, private accredited and private central labs for a steadily growing number of different analyses, including comprehensive genomic profiling^22^. Collectively, these data clearly indicate that there is an urgent need for the NHS to recognize the pivotal role of pathology units in the pre-analytical phases of complex molecular analyses, also when these are externalized. Adequate resources and personnel are in fact necessary to minimize turnaroundtime (TAT) and to guarantee prompt treatment initiation, that should not exceed one month after surgery in eBC patients^18^.

Since time required for sample preparation, shipment, and centralized Oncotype^®^ analysis is, on average, 10–14 working days (Fig. 1), integrating multigene tests into the traditional workflow clearly represents a challenge. As multigene testing is usually proposed by breast units, which holds weekly, the interval between surgery and the initiation of multigene testing procedures ranges from 5 to 9 days, and it could be delayed of additional two days in ER + /HER2 IHC equivocal cases for which the breast unit require ISH analysis. In this scenario, the authors consider that reflex testing ordered by pathologist could be a tool to significantly shorten multigene assay TAT: in fact, since ER/PgR/HER2 status and Ki-67 labeling index are routinely determined in diagnostic core biopsies in most of the cases, all the criteria for prescribing multigene tests are available to pathologists soon after the completion of pTNM in surgical specimens. Reflex testing could therefore spare the time elapsing from the pathological report and the multidisciplinary team (MDT) discussion. Criteria for reflex testing should be carefully discussed by scientific societies and formally validated by national guidelines, and of course would not prevent multidisciplinary case by case discussion for further personalization. In a recent meeting held in Milan for discussing scientific and organizational issues related to multigene testing in Italian pathology units, including reimbursement for IHC characterization and proper funding of the additional workload, we propose that pathologists could order a multigene test for female ER + /HER2- early BC patients, aged <65 years and matching the following criteria:

- pT1 (>1 cm) or pT2, pN0 (pre- or post-menopausal) and pN1a (post-menopausal)
- G2/G3

- ER > = 30%
- Ki-67 20–40% OR PgR < 20%

**Fig. 1:**
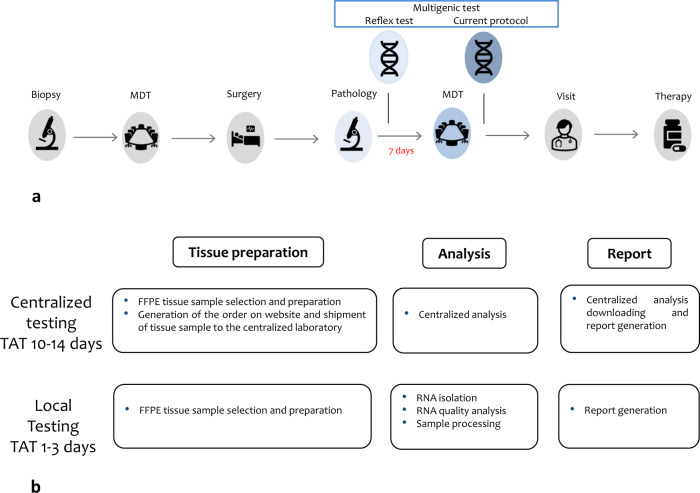
Workflow and turn-around-time (TAT) of breast cancer patients multigene testing. **a** A scheme of the current management, underlining the potential role of reflex testing actioned by pathologist in shortening TAT. **b** Detail of the pathology lab workload and TAT for outsourced and in house multigene testing. Third party material not used.

This approach would accelerate the decision-making process and favor timely testing with multigene assays for optimal adjuvant treatment. Patients not fulfilling the reflex test criteria will be discussed in the MDT meeting according to the established routine practice. This approach could lead to a slight increase of unnecessary testing volume, since a small fraction of patients could eventually refuse CHT regardless the risk score. To minimize this potential flaw, patients will be informed about the purpose and the actionability of the multigene tests and an informed consent will be obtained before surgery. In the next months, we will start a multicentric observational study to prospectively assess the validity of reflex testing in properly selecting patients for multigene analysis and in shortening TAT. Briefly, we will evaluate in six reference centers nationwide the concordance between reflex and breast unit criteria in selecting patients for multigene testing, as well as the validity of reflex testing in shortening TAT. Interestingly, a previous experience at Dana Farber with surgeon-initiated reflex Oncotype DX^®^ testing demonstrated a clinically significant reduction in wait times to chemotherapy decision making and initiation^23^.

In conclusion, multigene testing is an essential tool for properly treating ER + /HER2- eBC, that integrates traditional clinicopathological analyses and requires changes in the traditional workflow, also potentially including reflex testing by pathologists. Its accurate and widespread application depends on the implementation of reliable procedures within the pathology units of the NHS, for which proper staff and reagents funding is urgently needed.

## Reporting summary

Further information on research design is available in the Nature Research Reporting Summary linked to this article.

## Supplementary information

Reporting Summary
